# Polyanionic Candidate Microbicides Accelerate the Formation of Semen-Derived Amyloid Fibrils to Enhance HIV-1 Infection

**DOI:** 10.1371/journal.pone.0059777

**Published:** 2013-03-27

**Authors:** Suiyi Tan, Lu Lu, Lin Li, Jixiang Liu, Yelena Oksov, Hong Lu, Shibo Jiang, Shuwen Liu

**Affiliations:** 1 School of Pharmaceutical Sciences, Southern Medical University, Guangzhou, China; 2 Key Laboratory of Medical Molecular Virology of Ministries of Education & Health, Shanghai Medical College and Institute of Medical Microbiology, Fudan University, Shanghai, China; 3 Lindsley F. Kimball Research Institute, New York Blood Center, New York, New York, United States of America; University of Amsterdam, The Netherlands

## Abstract

Polyanionic candidate microbicides, including cellulose sulfate, carrageenan, PRO 2000, were proven ineffective in preventing HIV-1 transmission and even cellulose sulfate showed increased risk of HIV acquisition in the Phase III efficacy trials. Semen plays critical roles in HIV-1 sexual transmission. Specifically, amyloid fibrils formed by fragments of prostatic acidic phosphatase (PAP) in semen termed semen-derived enhancer of virus infection (SEVI) could drastically enhance HIV-1 infection. Here we investigated the interaction between polyanions and PAP248-286, a prototype peptide of SEVI, to understand the possible cause of polyanionic candidate microbicides to fail in clinical trials. We found anionic polymers could efficiently promote SEVI fibril formation, most likely mediated by the natural electrostatic interaction between polyanions and PAP248-286, as revealed by acid native PAGE and Western blot. The overall anti-HIV-1 activity of polyanions in the presence or absence of PAP248-286 or semen was evaluated. In the viral infection assay, the supernatants of polyanions/PAP248-286 or polyanions/semen mixtures containing the free, unbound polyanionic molecules showed a general reduction in antiviral efficacy, while the pellets containing amyloid fibrils formed by the polyanion-bound PAP248-286 showed aggravated enhancement of viral infection. Collectively, from the point of drug-host protein interaction, our study revealed that polyanions facilitate SEVI fibril formation to promote HIV-1 infection, thus highlighting a molecular mechanism underlying the failure of polyanions in clinical trials and the importance of drug-semen interaction in evaluating the anti-HIV-1 efficacy of candidate microbicides.

## Introduction

Heterosexual intercourse accounts for more than 80% of new HIV infection worldwide [1]. Microbicides, containing anti-HIV agents applied topically by women within vagina, hold great promise as a powerful women-initiated prevention method to stop HIV/AIDS transmission. Moreover, effective microbicides can be used for rectal intercourse by men who have sex with men (MSM), which is also becoming an increasing risk of HIV-1 acquisition recently [2], or women having anal intercourse [3], [4]. However, no candidate microbicide has proceeded from successful clinical trials to licensure, although 1% of tenofovir gel showed 39% efficacy among tested women in the recent microbicide trial [5]. Continual failure emphasizes our incomplete understanding about the molecular events that are occurring during sexual transmission and the biological elements involved in this process.

Anionic polymers have been considered as compelling candidate microbicides not only by their efficacy against HIV-1 infection [6] but also against a broad spectrum of sexually transmitted infection (STI) pathogens [7], [8]. The most mentioned polyanionic candidate microbicides include cellulose sulfate, carrageenan, naphthalene sulfonate (PRO 2000), cellulose acetate phthalate (CAP) and polystyrene sulfonate. Three of these have been advanced into Phase III clinical trials. Unfortunately, the clinical results were disappointing. One of the cellulose sulfate trials even showed higher HIV seroincidence in the cellulose sulfate arm [9], while another efficacy trial indicated no inhibitory effect of cellulose sulfate on the risk of HIV-1 transmission [10]. Efficacy trial of carrageenan demonstrated that carrageenan gel was safe, but lacked efficacy against HIV-1 transmission [11]. Similarly, PRO 2000 was not efficacious against vaginal HIV-1 transmission. Rate of new HIV-1 infection (incidence per 100 woman-years) among participants who used 0.5% PRO 2000 was 4.5% compared to that of 4.3% in the placebo arm, while HIV-1 infection rate was 4.7% in women administrated 2% PRO 2000 compared to that of 3.9% in placebo arm [12], [13]. Though the difference was not statistically significant (p = 0.239), use of 2% PRO 2000 gel was terminated at primary safety endpoint. It appeared that increasing dosage of PRO 2000 might implicate trends towards growing risk of HIV-1 transmission.

Basic steps uncover the failure of these polyanionic candidate microbicides have been made [14]–[17]. However, these results have placed greater focus on the participants or the antiviral agent alone. Besides these factors, we and others believe that other factors involving the host environment during sexual intercourse that could affect the efficacy of polyanion-based microbicides may also contribute to the failure of these polyanions in clinical trials [18].

Semen is one such important factor in the host environment during the sexual transmission. In the *in vitro* studies, candidate microbicides showing potent activities against HIV-1 infection were introduced in buffer [19]. But buffer solution does not compare with the true host environment during heterosexual transmission where drugs encounter female genital tract secretions and semen. Although semen has been documented to reduce the antiviral efficacy of candidate microbicides [20], [21], limited mechanistic research has been done to elucidate how semen interferes with anti-viral activity of candidate microbicides.

Semen-derived amyloid fibrils can extraordinarily enhance HIV-1 infection [22]. These amyloid fibrils, termed semen-derived enhancer of virus infection (SEVI), compose of fragments of the prostatic acidic phosphatase (PAP, predominant form PAP248-286) in semen. By their cationic property to capture virus and promote viral attachment to the cells, SEVI can enhance the infectious virus titer by several orders of magnitude [22], [23]. SEVI plays a critical role in sexual transmission of HIV-1.

The chemical structures of polyanionic candidate microbicide are strikingly similar with those of glycosaminoglcans (GAGs), a class of special linear polysaccharides that compose the polysaccharide-protein conjugates called proteoglycans. GAGs have been proven to play an active role in promoting fibrillogenesis *in vitro* for several proteins or peptides [24]–[30]. Therefore, we hypothesized the polyanionic candidate microbicide might act like GAGs to promote the formation of semen derived amyloid fibrils, leading to the increased risk of viral transmission in semen. Moreover, the promotion of fibrillogenesis by GAGs is mediated by the specific interaction between the peptide/protein and the polysaccharide [26], [27], [31]. We also intended to determine whether the promotion of SEVI fibril formation by polyanions is mediated by specific interaction between them. The objectives of this study were to examine the possible interaction between polyanions and seminal proteins that might underlie the failure of the clinical trial of polyanionic candidate microbicides.

## Materials and Methods

### Materials

λ-Carrageenan, Cellulose acetate phthalate (CAP), methyl cellulose, Thioflavin T, Congo Red, XTT [2,3-bis (2-methoxy-4-nitro-5-sulfophenyl)-5-(phenylamino) carbonyl-2H-tetrazolium hydroxide] were purchased from Sigma (St. Louis, MO). Cellulose sulfate was bought from Acros Organic, (Piscataway, NJ). Poly(styrene-4-sulfonate) was obtained from Polysciences Inc. Each polyanionic agent was dissolved in distilled water and divided in aliquots and kept at −20°C. Each aliquot was used only once after thawing. PAP248-286 (a prototype peptide of SEVI) was synthesized by GL Biochem (Shanghai, China). Lyophilized peptide (>95% purity) was dissolved in phosphate buffered saline (PBS) at a concentration of 10 mg/ml and stored at −20°C. Aliquots were rapidly defrosted and used as peptide monomer. The remainder was discarded. Otherwise, PAP248-286 fibril (SEVI) formation was promoted by agitation at 37°C for 1–5 days at 1200 rpm with an Eppendorf Mixmate. Polyclonal rabbit antibody against PAP248-286 was produced by AbMax Biotechnology Co., Ltd. (Beijing, China). Semen (SE) samples were collected from healthy lab members with written informed consent. The study was approved by the Human Ethics Committee of Southern Medical University, Guangzhou, China. SE samples were also purchased from Lee. BioSolutions. Inc. (St. Louis, Missouri, MO). Ejaculates were allowed to liquefy for 30 min at room temperature. Seminal fluid (SE-F), representing the cell free supernatant of SE, was obtained by centrifugation of 1 ml SE for 15 min at 10,000 rpm and stored in 1 ml aliquots at −20°C. Samples were used once and the remaining was discarded. MT-2 cells, TZM-bl cells, laboratory-adapted HIV-1 strains, anti-p24 monoclonal antibody (183-12H-5C), were obtained from the National Institutes of Health AIDS Research and Reference Reagent Program. Plasmids of CXCR4-tropic NL4-3 and CCR5-tropic 92th014.12 infectious clones were kindly provided by Jan Münch of Ulm University, Ulm, Baden-Württemberg, Germany.

### Monitor of PAP248-286 Aggregation

Fibril formation of PAP248-286 was monitored by Thioflavin T staining and Congo red staining as previously described [22]. Briefly, fibril formation was promoted by incubating 2 mg/ml PAP248-286 in the presence or absence of 30 µg/ml polyanions by agitation at 1200 rpm at 37°C with an Eppendorf Mixmate. At different incubation times after the initiation of the aggregation reaction, aliquots were withdrawn from each sample for the following tests. For Thioflavin T staining, 5 µl samples were mixed with 95 µl 50 µM Thioflavin T solution. Fluorescence emission was measured with a fluorescence plate reader (Infinite M1000, Tecan) as previously described [22]. For Congo red staining, formation of amyloid structures was routinely monitored by mixing 10 µl aliquots of the individual reaction batch with 90 µl of Congo red solution (Sigma). The solution was incubated at room temperature for 30 min and centrifuged for 15 min at 13000 rpm. The pellets were dissolved in 50 µl DMSO and fibril formation was determined at OD 490–650 nm using an ELISA reader (Infinite M1000, Tecan). All the samples were blank subtracted. No amyloid seeds were added to the mixture of the PAP248-286 and a polyanion before or during the formation of the amyloid fibrils.

### Analysis of β-Sheet Structure by Circular Dichroism (CD)

Aliquots of the sample undergoing aggregation were withdrawn at regular time intervals to detect the β-sheet structures by CD. CD spectra were recorded on Jasco spectropolarimeter (Model J-715, Jasco Inc., Japan) at 20°C. Spectra were collected from 200 to 260 nm, with a 1 nm step, 1-nm bandwidth and 20-s collection time per step. Each spectrum was recorded as the average of two scans and was blank-subtracted. The final peptide concentration was 120 µg/ml.

### Analysis of Amyloid Fibrils by Electron Microscopy

To observe the shape of amyloid fibrils by electron microscopy, peptide suspension (500 µg/ml) at different time points were adsorbed for 2 min onto 200-mesh carbon-coated copper grids (Electron Microscopy Sciences, Hatfield, PA). Grids were subsequently stained with 2% aqueous uranyl acetate (Electron Microscopy Sciences) for 60 s. Fibrils were visualized with a Tecnai 12 (Philips/FEI, USA) transmission electron microscope.

### Acidic Native Polyacrylamide Gel Electrophoresis and Western Blot analyses

10% Polyacrylamide continuous native gels were prepared as described elsewhere [32]. PAP248-286 monomers (300 µg/ml) were incubated with serially diluted solution of polyanionic agents at 37°C for 30 min. Then the samples were centrifuged at 5000 rpm for 3 min and the supernatant was mixed with equal volume of 20% glycerol prior to loading. Gels were either stained with Coomassie blue (loaded with 30 µl sample) or transferred by Western blot (loaded with 10 µl sample) to nitrocellulose membranes (Bio-Rad Laboratories, Hercules, CA). Membranes were probed with PAP248-286 polyclonal rabbit primary antibody and then with goat anti-rabbit secondary antibody conjugated to horseradish peroxidase. Signals were visualized with ECL Western blot Substrate Reagents and Amersham Hyperfilm (GE Healthcare, Piscataway, NJ).

### HIV-1 Infection Assays

To observe the effect of polyanions on infection by HIV -1_IIIB_ (subtype B, X4) and HIV-1_Bal_ (subtype B, R5) in the presence or absence of PAP248-286, two folds serially diluted polyanions were incubated with 100 µg/ml PAP248-286 in PBS or PBS only at 37°C for 1 h. The mixture was centrifuged at 5,000 rpm for 3 min. The inhibitory activities of the unbounded polyanions in the supernatants on infection of laboratory adapted HIV-1 strains were determined as previously described [33], [34]. For inhibition of infection by HIV-1 strain IIIB, 30 µl supernatants were mixed with HIV-1_IIIB_ at 100 TCID_50_ (50% tissue culture infective dose) and then 1×10^5^/ml MT-2 cells in RPMI medium 1640 containing 10% FBS were added to a final 200 µl of culture medium. The culture supernatants were replaced with fresh medium the next morning. On the fourth day post-infection, 100 µl of culture supernatants were collected from each well and mixed with equal volumes of 5% Triton X-100 for determination of p24 antigen by ELISA as described elsewhere [35]. For inhibition of infection by the HIV-1 strain Bal, 30 µl supernatants were mixed with HIV-1_Bal_ at 100 TCID_50_ and then 1×10^5^/ml TZM-b1 cells in DMEM medium containing 10% FBS were added to a final 200 µl of culture medium. Luciferase activity was measured 48 h postinfection using a luciferase assay kit (Promega, Madison, WI) by Ultra 384 luminometer (Tecan). The 50% inhibitory concentration (IC_50_) values were calculated by Calcusyn software, kindly provided by Dr. T. C. Chou at Sloan-Kettering Cancer Center (New York, NY).

To analyze the effect of polyanions on infection by HIV-1 in the presence of seminal fluid (SE-F), SE-F was incubated with equal volume of polyanions at graded concentrations at 37°C for 1 h. Then the mixtures were centrifuged at 5,000 rpm for 3 min. The supernatant was incubated with HIV-1_IIIB_ or HIV-1_Bal_ at 100 TCID_50_ respectively. 10^4^ TZM-bl cells were added. Luciferase activity was measured as indicated above. To avoid the cytotoxicity of SE-F, SE-F was used at 1∶500 dilution in these experiments.

To determine the enhancing effect of SEVI (fibrillar form of PAP248-286) formed in the absence (PBS only) or the presence of various polyanions in PBS on infection by HIV-1 R5-tropic 92th014.12 virus or X4-tropic NL4-3 virus, 60 µl of HIV-1 was mixed with 60 µl of SEVI at graded concentration, respectively, at room temperature for 10 min, 100 µl mixture was added to 1×10^4^ TZM-b1 cells, which had been cultured overnight. After a 3-h incubation period, unbound viruses were removed and cells were replaced with fresh DMEM containing 10% FBS. Luciferase activity was measured 3 days postinfection as described above.

The enhancing effect of the pellet portion of SE-F in the presence of various polyanions on infection by HIV-1 R5- or X4-tropic clone virus was determined as described above, except for the pretreatment of the samples. 90 µl SE-F was mixed with 10 µl polyanions at graded concentration. Mixtures were agitated at 1,200 rpm at 37°C for 5 h. SE-F was then centrifuged at 5,000 rpm for 3 min. Supernatants were discarded and the pellets were re-dissolved in 100 µl medium. Five µl of the suspension were mixed with 55 µl medium, which was then mixed with HIV-1 R5- or X4-tropic clone virus respectively. Mixtures were added to the TZM-b1 cells and infection was determined as indicated above.

**Figure 1:**
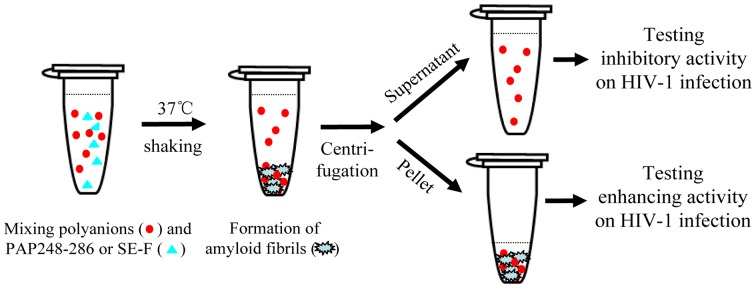
A brief schematic of the assays for testing the effect of polyanions on HIV-1 infection in the presence of PAP248-286 or SE-F. Polyanions at graded concentration was mixed with an indicated concentration of PAP248-286 or SE-F at an indicated interval with agitation as described in the “Materials and Methods”. After centrifugation, the supernatants were collected for testing the inhibitory activity of the unbounded polyanions on HIV-1 infection. The pellets containing the PAP248-286 (SEVI) - or SE-F-derived amyloid fibrils were re-suspended in 100 µl medium for testing the enhancing effect on HIV-1 infection.

Figure 1 showed a brief schematic of the above experiments for testing the effect of polyanions on HIV-1 infection in the presence of PAP248-286 or SE-F.

### Cytotoxicity Assay

The potential cytotoxic effect of SE-F and polyanions was detected by XTT assay as previously described [35]. Briefly, 100 µl of the tested polyanions at 50 µg/ml and SE-F at 1∶500 dilution or 100 µl of seminal pellet suspension (1∶200) were added to 100 µl of cells (5×10^5^/ml) in wells of 96-well plates, respectively. After incubation at 37°C for 2 days, 50 µl of XTT solution (1 mg/ml) containing 0.02 µM phenazine methosulfate (PMS) was added. After 4 h, the absorbance at 450 nm (A450) was measure with an ELISA reader.

## Results

### Polyanionic Candidate Microbicides Accelerate the Kinetics of PAP248-286 Aggregation

**Figure 2:**
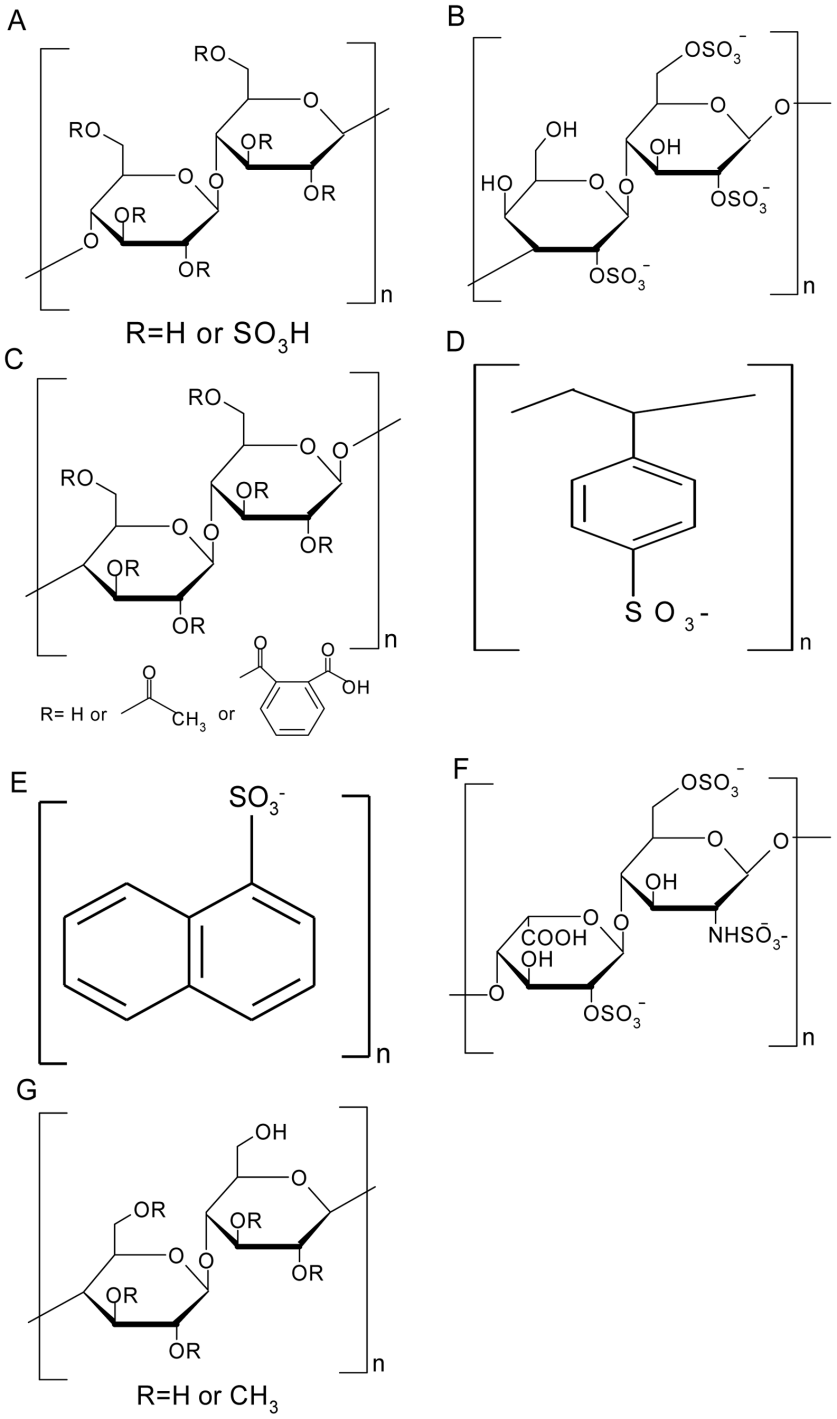
Chemical structures of polyanion-based microbicides and GAGs. Chemical structures of the molecules include cellulose sulfate (A), λ-carrageenan (B), CAP (C), polystyrene sulfonate (D), PRO 2000 (E), heparin sulfate (F) and methyl cellulose (G).

Four anionic polymers were selected as our study objects (Fig. 2A–D). The core structure of these polyanions is composed of a carbon backbone, such as repeating disaccharide unit, benzenoid ring and naphthalic ring, and negatively charged moiety distributed along the carbon backbone (Fig. 2). The sulfate group contributes to the negative charge in most cases. Cellulose sulfate, carrageenan, and CAP (Fig. 2A–C) [36] consist of a repeating disaccharide unit linked to the sulfate side chains or acetate group, closely resembling natural GAGs, such as heparin sulfate (Fig. 2F). Polystyrene sulfonate (Fig. 2D) has been in preclinical trial and shares a structure similar to PRO 2000 (Fig. 1E) [37], [38].

**Figure 3:**
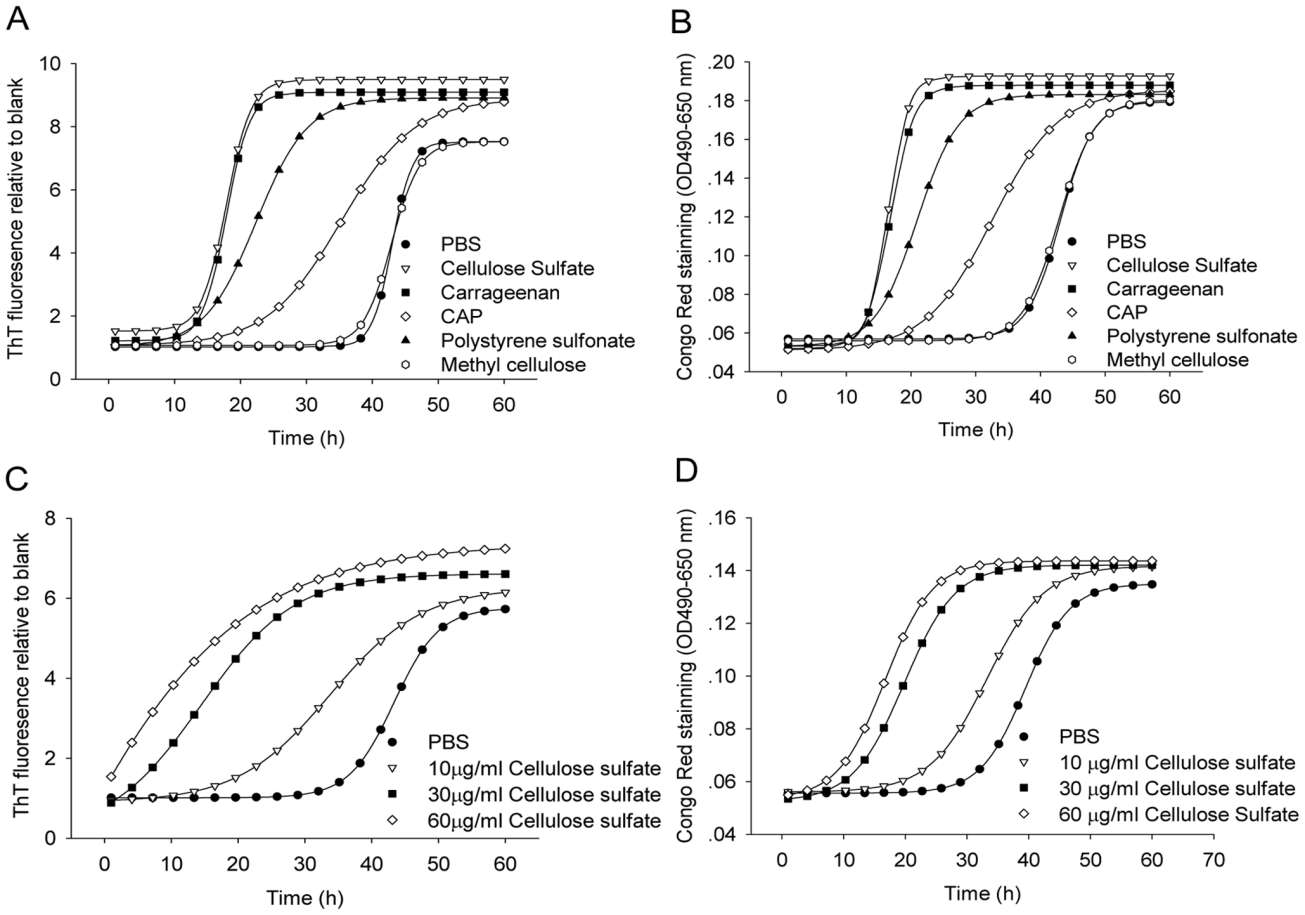
Time courses of PAP248-286 aggregation in the absence or presence of polyanionic candidate microbicides. 2 mg/ml PAP248-286 was agitated at 37°C and 1200 rpm in the presence or absence of various polyanions (30 µg/ml). The status of peptide aggregation is monitored by Thioflavin T staining (A) or Congo red staining (B). The facilitation of the fibrillogenesis of PAP248-286 mediated by cellulose sulfate is dose-dependant as revealed by Thioflavin T staining (C) and Congo red staining (D). The data presented were the median values obtained from one experiment performed in triplicate. Experiments were repeated once that yielded similar trends.

We first determined whether these polyanions exhibited a biological function to facilitate SEVI formation. Fibrilization assays were performed with PAP248-286 in the presence or absence of polyanions by two amyloid fibril specific probes, thioflavin T and Congo Red. These two dyes can form a complex with amyloid fibrils respectively and result in increased fluorescence intensity or optical absorbance proportional to the amount of fibrils. As we could see the time-dependent trend of PAP248-286 aggregation in the presence or absence of candidate microbicides in Figure 3, all the polyanionic microbicides showed obvious promotion of SEVI fibril formation by thioflavin T staining (Fig. 3A) and Congo Red staining (Fig. 3B). In the absence of polyanions, we observed typical nucleation-dependent polymerization process of PAP248-286, characterized by a sigmoidal time course with a lag phase of around 40 hours. The substantial loss of the lag-phase in the presence of polyanions suggested a faster fibril formation process. The length of the apparent lag phase was brought forward to approximate 12 hours in the presence of cellulose sulfate, carrageenan and polystyrene sulfonate. However, CAP promoted SEVI amyloid fibril formation to a lesser extent with a lag phase around 20 hours. Notably, polyanions also induced a progressive increase in the magnitude of the fluorescence or absorbance plateau value, reflecting a greater yield of fibril formation compared to the peptide alone. Specifically, methyl cellulose, a neutral polymer (Fig. 2G), was used as the negative control. Methyl cellulose exerted no influence on SEVI fibril growing curve (Fig. 3A–B), indicating an essential role of the negative moiety in promoting amyloid fibril formation by polymers. Moreover, the enhancement of SEVI fibril formation in the presence of cellulose sulfate occurred in a dose-dependent manner (Fig. 3C and 3D).

### Polyanionic Candidate Microbicides Promote β-sheet Formation of PAP248-286

**Figure 4:**
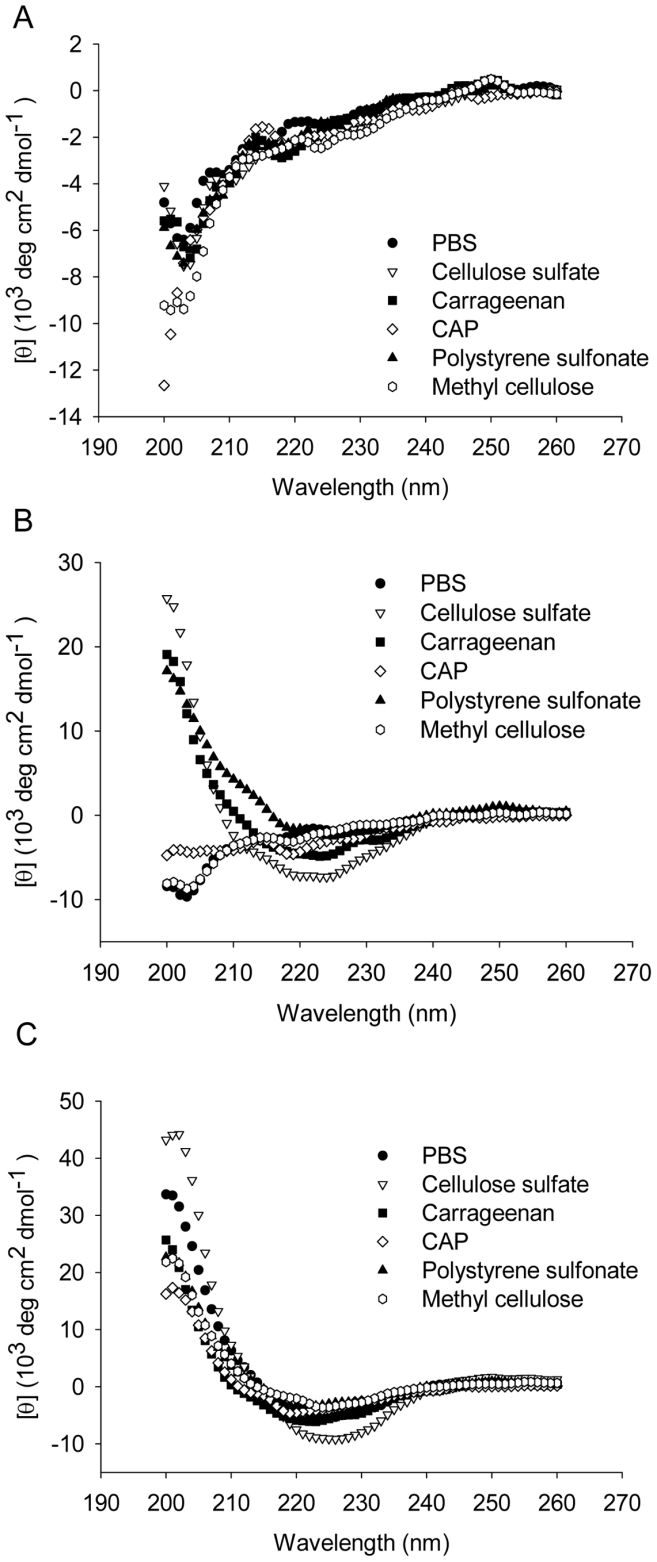
CD spectroscopic analysis of the β-sheet structure of PAP248-286. The spectra of PAP248-286 in the presence or absence of polyanions were calculated by subtracting the spectra of PBS or polyanions from those of PAP248-286 alone or PAP248-286+polyanions respectively. A, t = 12 h; B, t = 36 h; C, t = 60 h. The final peptide concentration of each sample was 120 µg/ml. The experiment was repeated once and similar result was obtained.

Amyloid fibril formation is characterized by the β-sheet aggregation. We applied CD as a function of time to investigate the conformation changes associated with the aggregation of PAP248-286 with or without polyanionic candidate microbicides. Spectra were recorded at different time points soon after the onset of the aggregation reaction. At 12 h, all spectra remained much the same with minimum centered at around 200 nm, showing a characteristic of random coiled structure (Fig. 4A). However, at the time of 36 h, the spectra of samples in the presence of cellulose sulfate, carrageenan and polystyrene sulfonate underwent great transition from random coil to β-sheet formation, manifesting a minimum absorbance at 222 nm (Fig. 4B). The spectra of sample with CAP gave rise to a minimum at 222 nm whereas that at 200 nm gradually disappeared as aggregation proceeded. The spectra of peptide control and peptide with methyl cellulose continued to be typical random coiled structure. The β-sheet transition has not yet occurred. At the time point of 60 h, all the samples displayed typical β-sheet structure (Fig. 4C). The CD result indicated that polyanionic candidate microbicides accelerated the SEVI amyloid fibril formation by promoting the structural transition of PAP248-286 from random coil to a cross β-sheet structure.

### Polyanion-based Microbicides Facilitate the Process of PAP248-286 Fibril Formation

**Figure 5:**
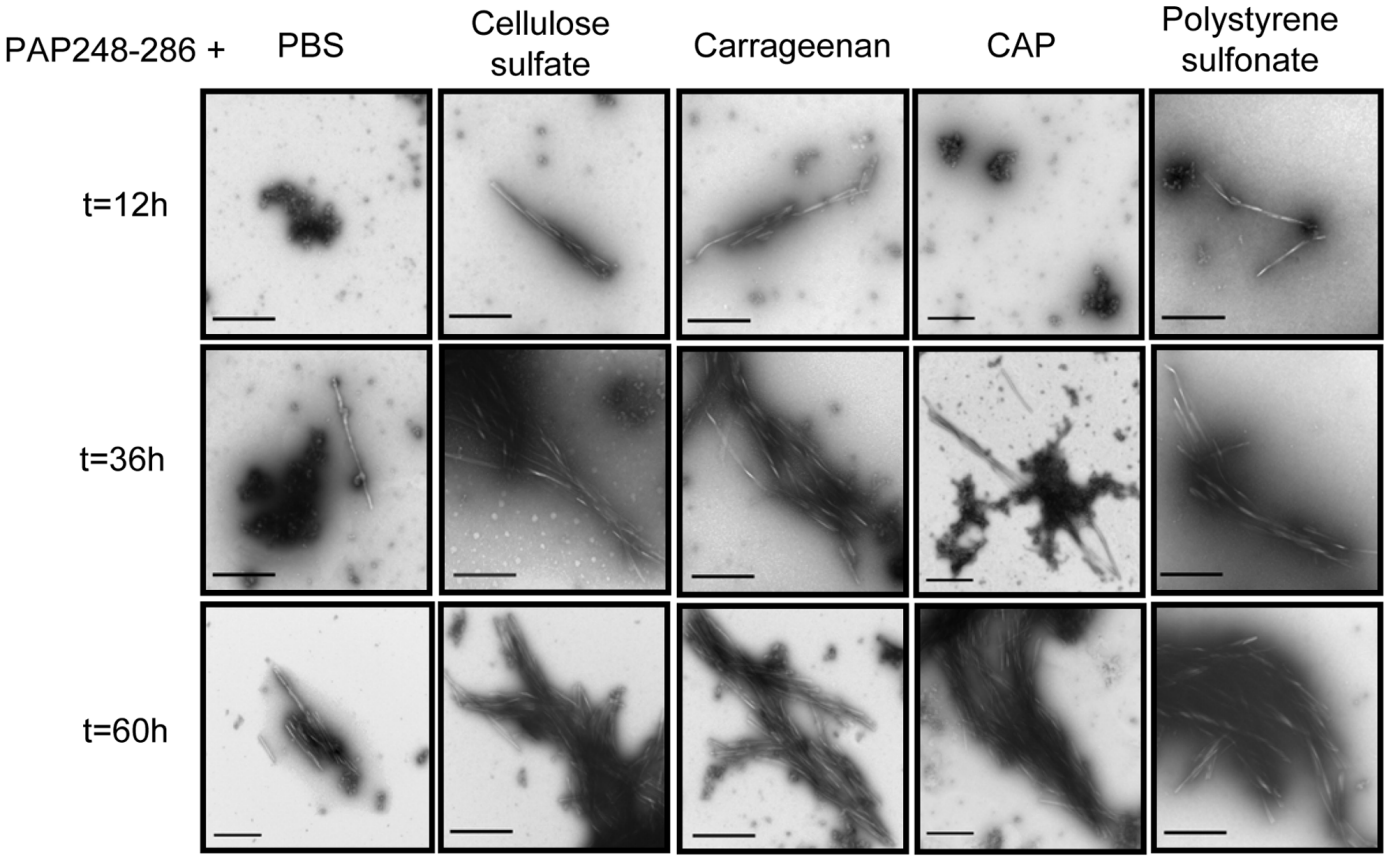
Transmission electron microscopic analysis of the fibrillar shape of PAP248-286. 2 mg/ml PAP248-286 was incubated in the presence or absence of 30 µg/ml polyanions. The amyloid fibril shapes were visualized by negative-stain EM at different time points (12 h, 36 h, 60 h). Images are shown relative to a scale bar of 500 nm (magnification: 18,500×).

With transmission electron microcope, we could see the facilitated process by characterizing the morphology of the PAP248-286 aggregates following the time course of fibril formation (Fig. 5). At the time point of 12 h, we found only granular species with peptide control and sample in the presence of CAP, instead of any fibril structures. However, small pieces of fibril-like protofibrils could be detected with PAP248-286 in the presence of cellulose sulfate, carrageenan and polystyrene sulfonate. At the time point of 36 h, all polyanion-treated samples aggregated further to form higher ordered structure and gave rise to mature fibrils while only small piece of fibril-like structure could be detected with PAP248-286 control. As time proceeded to 60 h, we could see bundles of mature fibrils with PAP248-286 in the presence of polyanions while mature fibrils just appeared with PAP248-286 in the absence of polyanions.

### Polyanion-based Microbicides Directly Interact with PAP248-286 by Electrostatic Interaction

**Figure 6:**
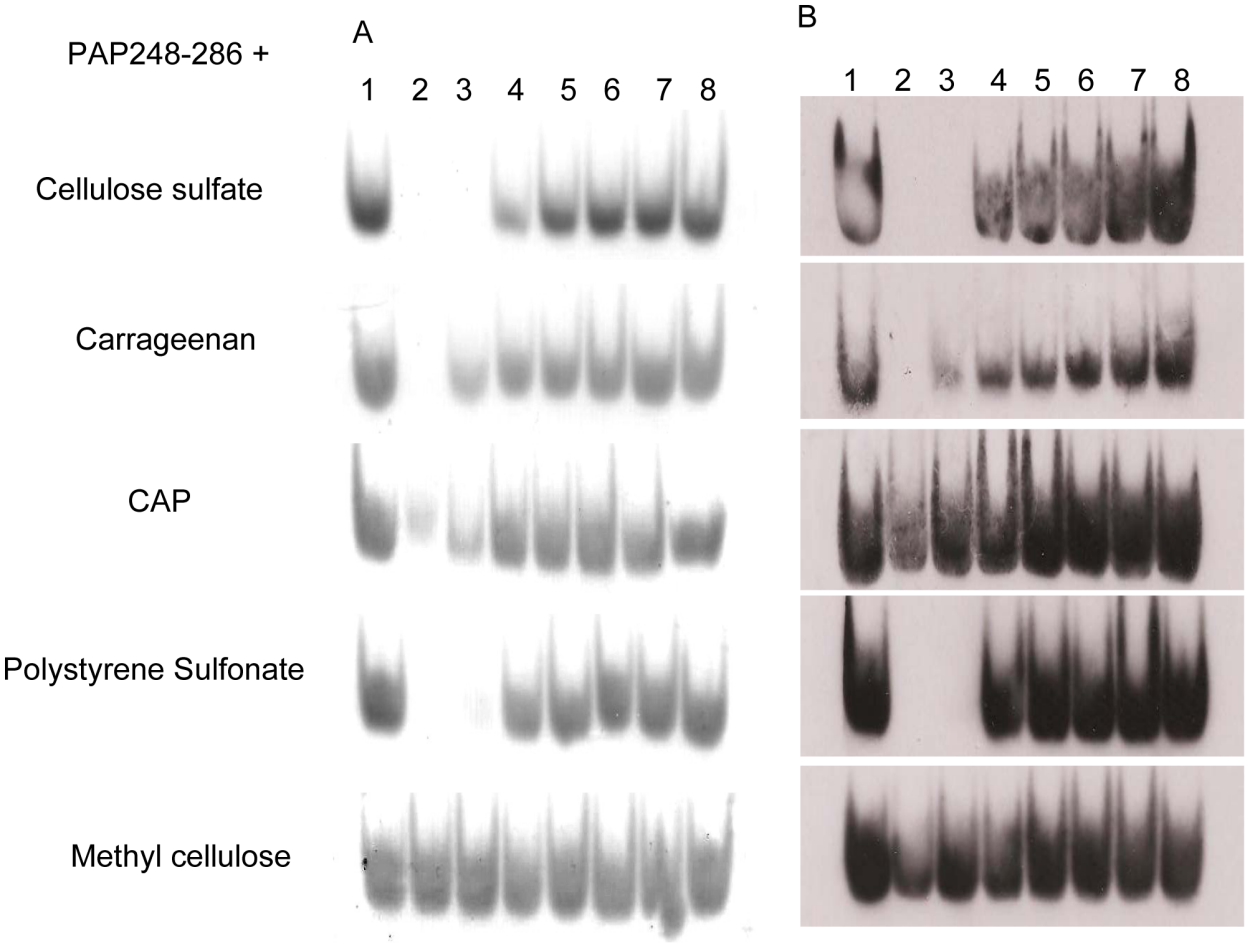
Interaction between PAP248-286 and polyanionic candidate microbicides. 300 µg/ml PAP248-286 was incubated with various polyanions at graded concentration and then the remaining peptides in the supernatants were electrophoresed on 10% native polyacrylamide continuous gels. Gels were either stained with Coomassie Blue (A) or subjected to immunoblotting with anti-PAP248-286 polyclonal antibody (B). The concentrations of polyanions were 0, 300, 150, 75, 37.5, 18.75, 9.375, 4.7 µg/ml (from lane 1 to lane 8), respectively.

As we know, polyanions are negatively charged due to the sulfate moiety while PAP248-286 is positively charged [23]. Whether the interaction exists between the two oppositely charged molecules might account for polyanions mediated enhancement of SEVI fibrils formation and the clinical failure of polyanionic microbicides? Thus, acid native gels were applied to analyze the potential interaction between polyanions and PAP248-286 by determining the remaining free PAP248-286 in the supernatant after incubation of the peptide and various anionic polymers. Samples were loaded in duplicate, electrophoresed in parallel, and then the gels were either stained with Coomassie Blue or visualized by Western blot with rabbit polyclonal antibody against PAP248-286. Both gels hardly revealed any residual peptide in the supernatant when peptide was incubated with polyanions, at a ratio of 1∶1 (w/w; Fig. 6, lane 2). In response to the addition of increasing amount of polyanions, a gradual reduction of peptide in the supernatant was detected after centrifugation, suggesting PAP248-286 that was initially present in solution formed aggregated complex with polyanions. However, no difference was observed in the quantity of PAP248-286 in the supernatant when peptide was incubated with methyl cellulose (Fig. 6), implying PAP248-286 did not interact with methyl cellulose.

### Evaluation of the Effect of Polyanionic Candidate Microbicides in the Presence of PAP248-286 or SE-F on Viral Infection

Based on the above findings, we hypothesize two root causes underlie the failure of polyanions at clinical trial. First, polyanions facilitated SEVI fibril formation, increasing the chance of viral infection in semen. Second, the specific interaction between polyanions and PAP248-286 might lower the concentration of free drug, thus weakening the anti-HIV activities of the polyanions. Therefore, in the case of the polyanions/PAP248-286 mixture or polyanions/SE-F mixture, the supernatant presents evidence that the free drugs might show decreased anti-HIV activities, while the pellet portion served as evidence that the potential aggregates might display an augmented ability to enhance HIV-1 infection. We first evaluated the anti-HIV activity of polyanions in the presence of 100 µg/ml PAP248-286 and SE-F. Inhibition of HIV-1 IIIB and HIV-1 BaL infection by the supernatant of polyanionic candidate microbicides in the absence or presence of PAP248-286 or SE-F was determined. In the infection assays, SE-F was diluted to 500 folds and the culture supernatants were replaced with fresh medium 12 h later to avoid cytotoxicity to the target cells. The data in Table 1 indicated a general decreased anti-HIV-1 activity of polyanions by 10–15 folds in the presence of 100 µg/ml PAP248-286. Results from Table 2 showed that the presence of SE-F led to an increase in the calculated IC_50_ of polyanionic candidate microbicides against laboratory-adapted HIV-1 strains by 7–20 folds.

**Table 1:** Effect of PAP248-286 on the antiviral activity of polyanionic candidate microbicides against laboratory-adapted HIV-1 strains^a^.

Polyanions	IC_50_ (µg/ml) for inhibiting HIV-1_Bal_ (R5) infection			IC_50_ (µg/ml) for inhibiting HIV-1_IIIB_ (X4) infection		
	+ PBS only	+ PAP248- 286 in PBS	Fold of increased	+ PBS only	+ PAP248 -286 in PBS	Fold of increased
Cellulose sulfate	0.92±0.07	9.26±0.10	9.1	0.73±0.07	7.50±0.07	9.3
Carrageenan	0.58±0.02	6.21±0.99	9.7	0.68±0.03	6.47±0.01	8.5
CAP	1.29±0.06	10.78±0.02	7.4	0.85±0.11	10.10±0.02	10.9
Polystyrene sulfonate	0.64±0.08	10.14±1.22	14.8	0.77±0.02	10.96±0.75	13.2

a IC_50_s are means ± standard deviations (n = 3).

**Table 2:** Effect of SE-F on the antiviral activity of polyanionic candidate microbicides against laboratory-adapted HIV-1 strains^a^.

Polyanions	IC_50_ (µg/ml) for inhibiting HIV-1_Bal_ (R5) infection			IC_50_ (µg/ml) for inhibiting HIV-1_IIIB_ (X4) infection		
	+ PBS only	+SE-F in PBS	Fold of increased	+ PBS only	+ SE-F in PBS	Fold of increased
Cellulose sulfate	1.33±0.22	12.05±1.47	8.1	0.85±0.07	11.26±1.08	12.2
Carrageenan	1.07±0.03	7.85±0.09	6.3	0.61±0.01	4.98±0.21	7.2
CAP	1.24±0.03	24.15±6.26	18.5	0.79±0.24	7.43±0.03	8.4
Polystyrene sulfonate	0.46±0.11	3.49±0.00	6.6	0.83±0.08	10.01±1.05	11.1

a IC_50_s are means ± standard deviations (n = 3).

**Figure 7:**
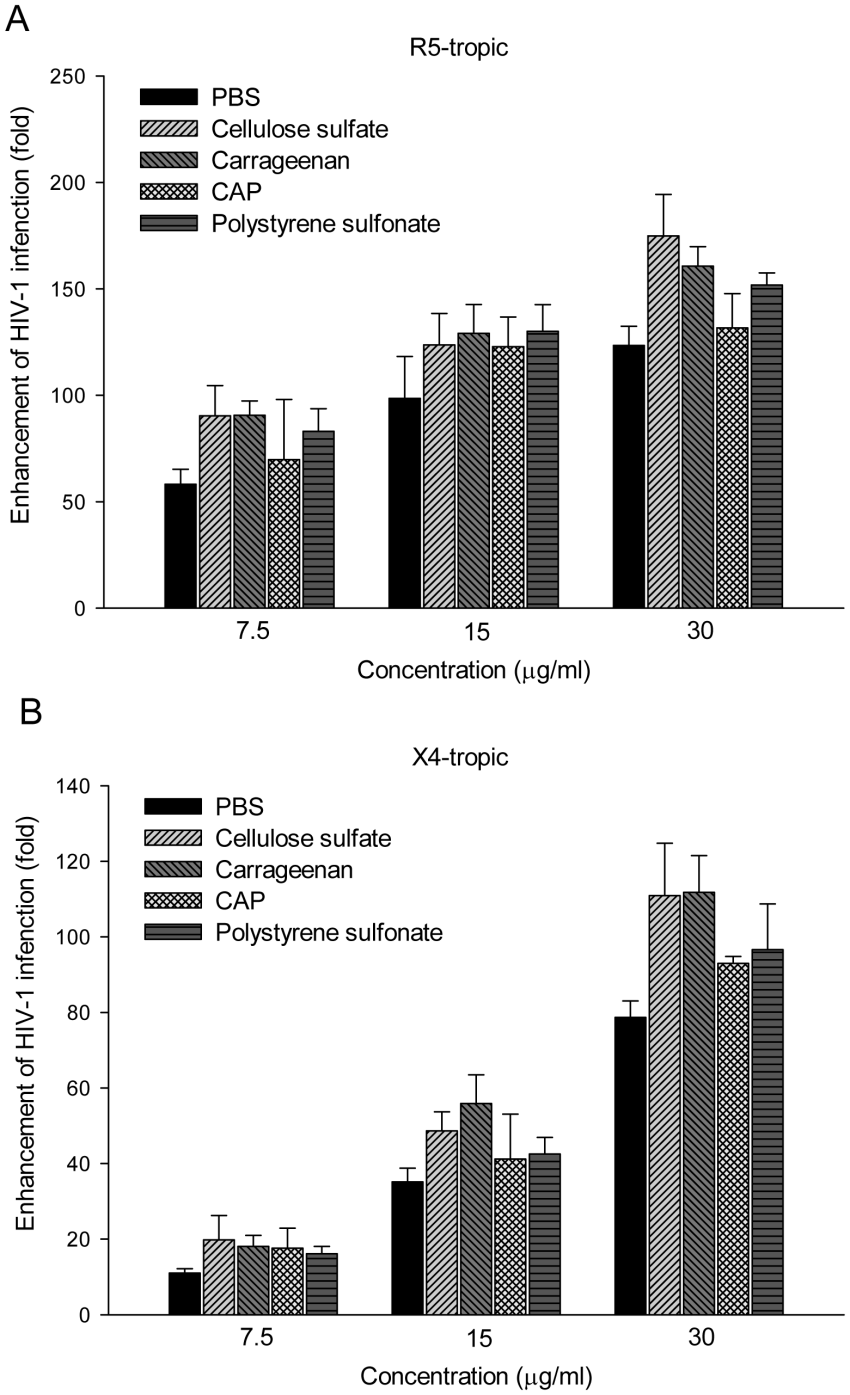
Enhancement of HIV-1 infection of SEVI in the presence of polyanions. Fibril formation of PAP248-286 was promoted by agitation in the presence of polyanions (30 µg/ml) or PBS for 60 h. PAP248-286 at different concentration were mixed with R5 tropic (A) and X4 tropic viruses (B), respectively. The mixtures were then added to TZM-b1 cells. After culture at 37°C for 3 h, the medium was replaced. Luciferase activity was detected 72 h later. Experiments were repeated once and similar results were obtained. Shown are average values (± standard deviations) of triplicate measurements of a representative experiment.

**Figure 8:**
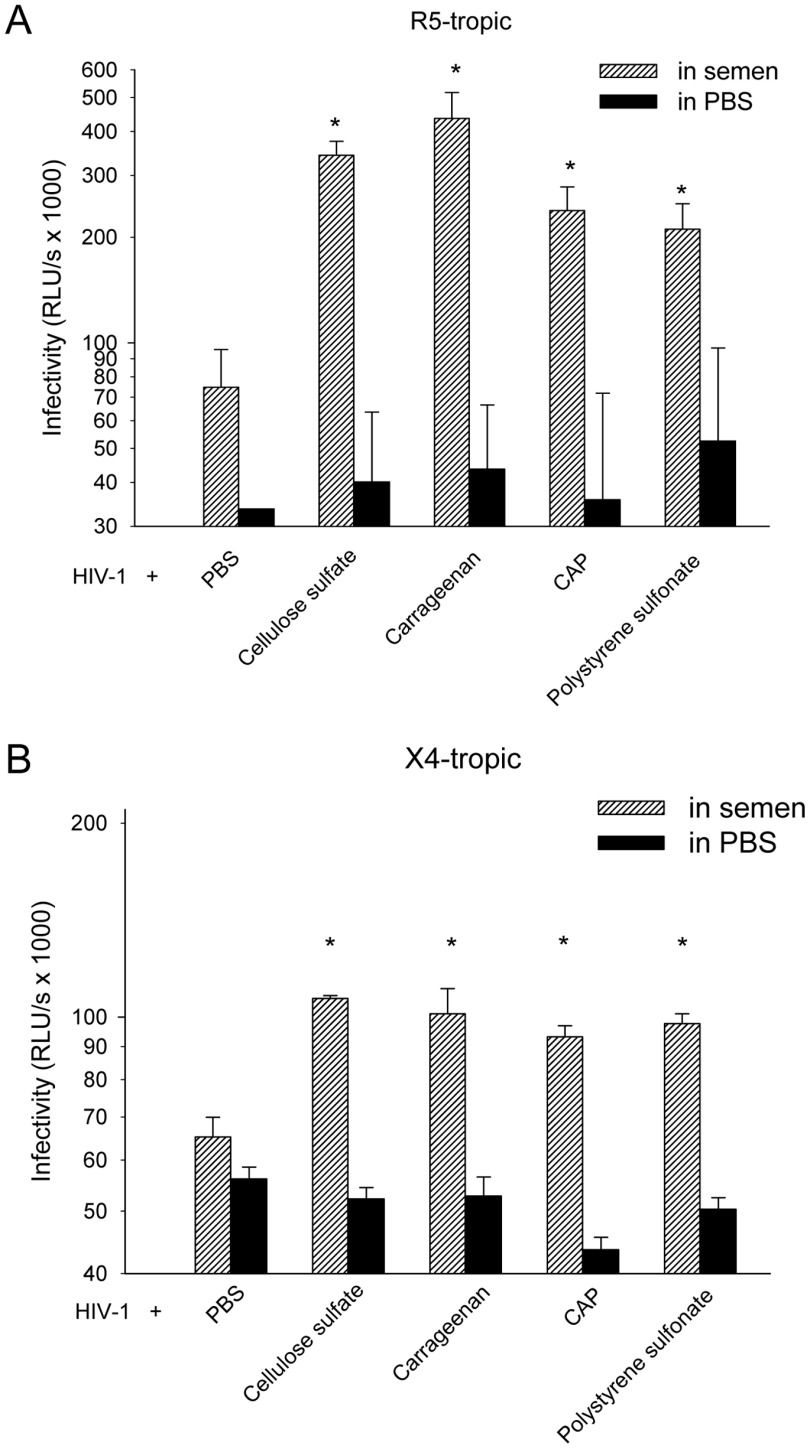
Enhancement of HIV-1 infection of SE-F in the presence of polyanions. SE-F or PBS (as a control) was agitated with various polyanions at graded concentration for 5 h. The pellet portion of the mixture was mixed with R5 tropic (A) and X4 tropic viruses (B), respectively. The mixtures were added to TZM-b1 cells. After culture at 37°C for 3 h, the medium was replaced. Luciferase activity was detected 72 h later. The experiment was repeated once and similar results were obtained. Shown are average values (± standard deviations) of triplicate measurements of a representative experiment. *vs. HIV-1 in SE, *P*<0.05 by *t*-test.

The abilities of SEVI, formed in the presence or absence of polyanions, to enhance HIV-1 infection were also demonstrated. SEVI alone could substantially enhance the infectiousness of X4 tropic HIV-1 and R5 tropic HIV-1 in single round infection assays (Fig. 7A and 7B). However, SEVI formed with polyanions showed an aggravated enhancement of viral infection compared to SEVI alone, which might be attributed to the increased amount of amyloid fibril in the presence polyanions. Next, we extended to find out whether the existence of polyanionic candidate microbicides might aggravate semen-mediated enhancement of HIV-1 infection. HIV-1 infectious clone were mixed with the pellet portion of seminal fluid in the presence or absence of polyanions at various concentration and the mixtures were used to infect TZM-bl indicator cells. Since low concentration of polyanions was reported to enhance HIV-1 infection [17], the residual molecules bound to the tube due to non-specific interaction might mask SE-F mediated enhancing effects. In order to circumvent this problem, we performed a drug control in which polyanions were introduced in PBS. As shown in Figures 8A and 8B, the residual of the polyanions had little effect on HIV-1 infection. The SE-F alone slightly enhanced infection of HIV-1 R5 virions by around one fold, while the addition of distinct polyanions induced aggravation of SE-F-mediated enhancement of HIV-1 infection (Fig. 8A). At the same time, these polyanions could also aggravate SE-F induced enhancement of X4 tropic HIV-1 infections (Fig. 8B). As a result of donor-to-donor differences, the extent of SE-F to enhance HIV-1 infection varies greatly [39]. Although our SE-F samples exerted weak enhancement of viral infection, the pellet of SE-F in the presence of polyanions induced greater increase in susceptibility to HIV-1 infection.

**Figure 9:**
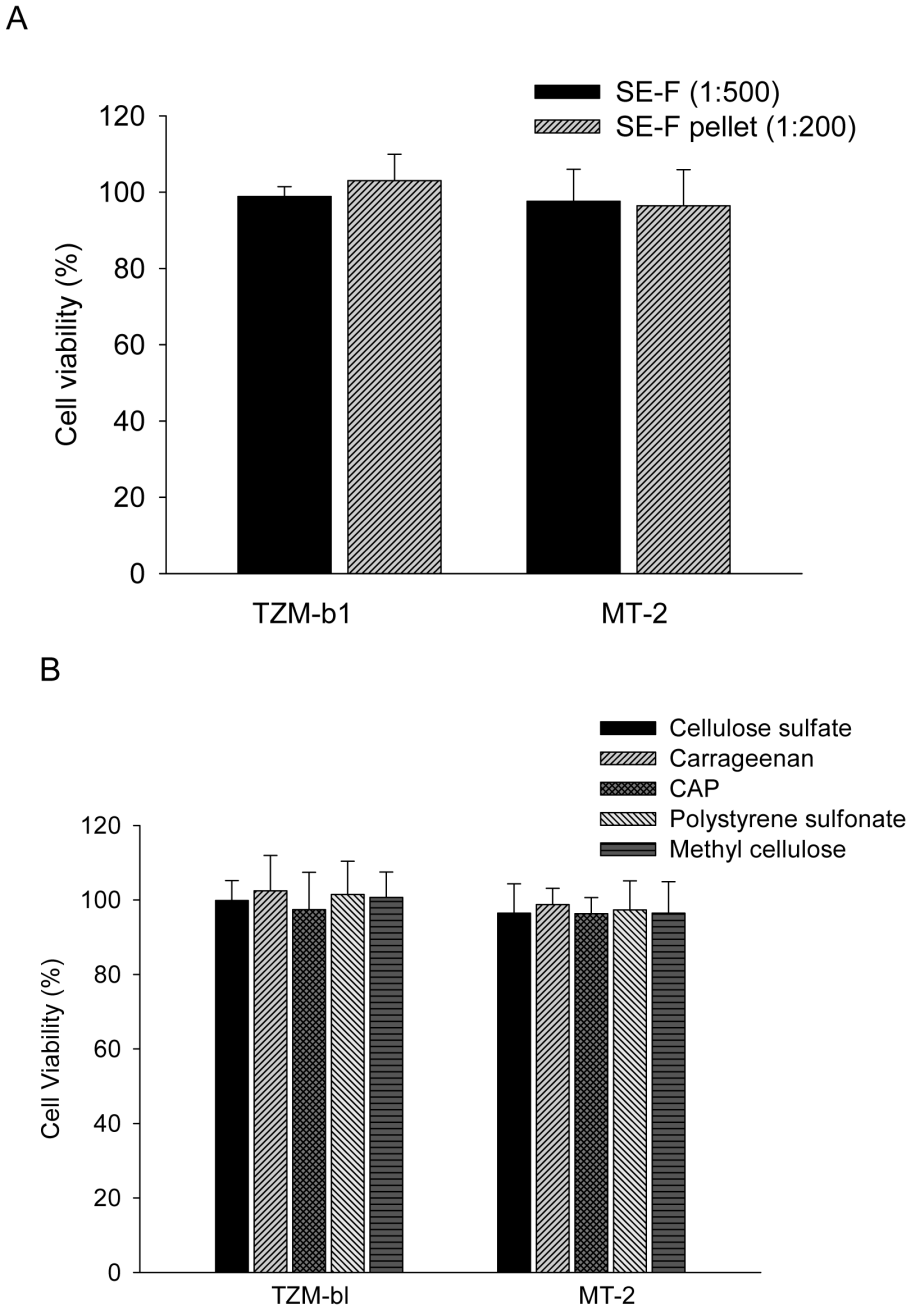
No significant cytotoxicity of SE-F and polyanions at the concentrations used in the present study. (**A**) Cytotoxicity of SE-F to different target cells. Seminal fluids (1∶500) and seminal pellet (1∶200) were incubated with TZM-bl cells or MT-2 cells respectively. After 48-h culture, cytotoxicity was determined by the XTT cell viability assay. The results were expressed as the mean of three different experiments. (**B**) Cytotoxicity of polyanions to different target cells. Various polyanions (50 µg/ml) were incubated with TZM-bl cells or MT-2 cells respectively. After 48-h culture, cytotoxicity was determined by the XTT cell viability assay. The results were expressed as the mean of three different experiments.

Using an XTT assay [35], we tested the potential cytotoxicity of SE-F and polyanions on TZM-bl and MT-2 cells, which were used in this study for studying the effects of SE-F and polyanions on HIV-1 infection. As shown in Fig. 9, SE-F at 1∶500 dilution in PBS, seminal pellet at 1∶200 dilution in PBS and polyanions at 50 µg/ml in PBS exhibited no significant cytotoxicity to these cell lines, suggesting that the enhanced HIV-1 infection is not resulted from the cytotoxicity of SE-F or polyanions at the concentrations that were tested in the present study.

## Discussion

A distinguished preclinical characterization of pharmacokinetics and pharmacodynamics of an active pharmaceutical agent provides a solid foundation for the success in applying for a new drug candidate. In respect of a microbicide, scientists have made rapid advances in the search on numerous candidates with remarkable efficacy against HIV-1 infection and suitable formulation [40], [41]. However, limited investigation has been done to analyze the potential host protein that might interfere with the effectiveness of a candidate microbicide. In view of the unique environment where the drug exerts its pharmaceutical effect during sexual intercourse, the biological interaction with seminal protein should be a significant determinant to evaluate the efficacy of a candidate microbicide.

Our results for the first time demonstrated that polyanionic candidate microbicides accelerated semen-derived amyloid fibril formation. In agreement with previous study [22], we confirmed that synthetic peptide PAP248-286 could form amyloid fibrils and substantially enhance the infectivity of R5-tropic and X4-tropic HIV-1 (Fig. 7). Surprisingly, exploring traditional methods to study amyloid fibrils [42], we obtained consistent results that the addition of various polyanionic candidate microbicides, caused a rapid aggregation of PAP248-286 into the beta sheet-rich amyloid fibrils, compared to a slow phase resulting from the normal aggregation (Fig. 3, 4, 5). Although various polyanions showed different abilities to facilitate SEVI fibril formation, the differences in the degree of sulfation, types of the negative group and molecular weight might have contributed to the distinct responses. The magnitude of the facilitation effect was correlated to the level of polyanions (Fig. 3C and 3D).

Furthermore, our data suggested that this promotion process of semen-derived amyloid fibril formation is mediated by the direct interaction of polyanions with PAP248-286. Owing to the naturally opposite charges, it is feasible that polyanions with negative charge interact with positively charged PAP248-286 by electrostatic interaction (Fig. 6). The common amyloid polymerization pathway proceeds through oligomeric intermediates to mature β sheet-rich fibrils [43]. Thus, the linear anionic polymers might serve as a template to concentrate and orient the peptide monomers or oligomers to associate by their electrostatic nature, thereby reducing the lag phase of fibrillization [44], [45].

In particular, it is been reported amyloid fibrils formed in the presence of polyanoins exhibit a higher resistance to trypsin and proteinase K proteolysis [30]. In our experiment, SE-F showed weak enhancement of viral infection, which might implicate that PAP-derived peptides were subjected to proteolytic activities within semen [46]. However, SE-F showed obvious enhancement of viral infection in the presence of polyanions. Therefore, SEVI formed in the presence of polyanions might be more resistant to proteolytic enzymes, which might give rise to their ability to enhance HIV-1 infection under physical conditions. Recently, apart from PAP-derived fragments, Roan and colleagues found that semen harbored various amyloidogenic peptides that could also enhance HIV-1 infection [47]. Thus, polyanionic candidate microbicides might promote non-PAP-derived amyloid fibril formation in semen as well.

The negatively charged groups of polyanionic candidate microbicides, which can selectively bind to the HIV-1 gp120 and inhibit the viral attachment to the cell surface, are essential for the anti-HIV activities [6], [48]. Apparently, polyanionic candidate microbicides might suffer loss of anti-HIV activity due to the neutralization of their negative charges by PAP248-286. Besides PAP248-286, semen and vaginal fluid, two main biological fluids during sexual intercourse, are rich in cationic peptides/proteins [49], [50]. It’s been estimated that the concentration of seminal protein could reach 170 mg/ml while cationic proteins could reach 5 mg/ml in semen [47], [50]. Polyanion-based microbicides might further shield from these cationic seminal or vaginal peptides. Even worse, this type of natural electrostatic interaction between polyanions and cationic peptides appears to be of low specificity but high affinity [31].

The effect of semen on HIV-1 sexual transmission remains controversial. Some reported that cationic peptides in semen exert anti-viral influence on HIV-1 infection while amyloid fibrils found in semen could drastically enhance HIV-1 infection [22], [50]. Hence, to examine the effects of polyanions with SE-F or PAP248-286 on HIV-1 infection, we tested the supernatant part and the pellet portion of the mixture of polyanions in the presence of PAP248-286 or SE-F against HIV-1 infection. In accordance with our proposed mechanism, the supernatant represents the soluble molecules display a general diminished anti-viral activity by 7–20 folds while the pellet portion on behalf of the aggregates showed an augmented enhancement of viral infection (Fig. 7 and 8, Table 1 and 2).

**Figure 10:**
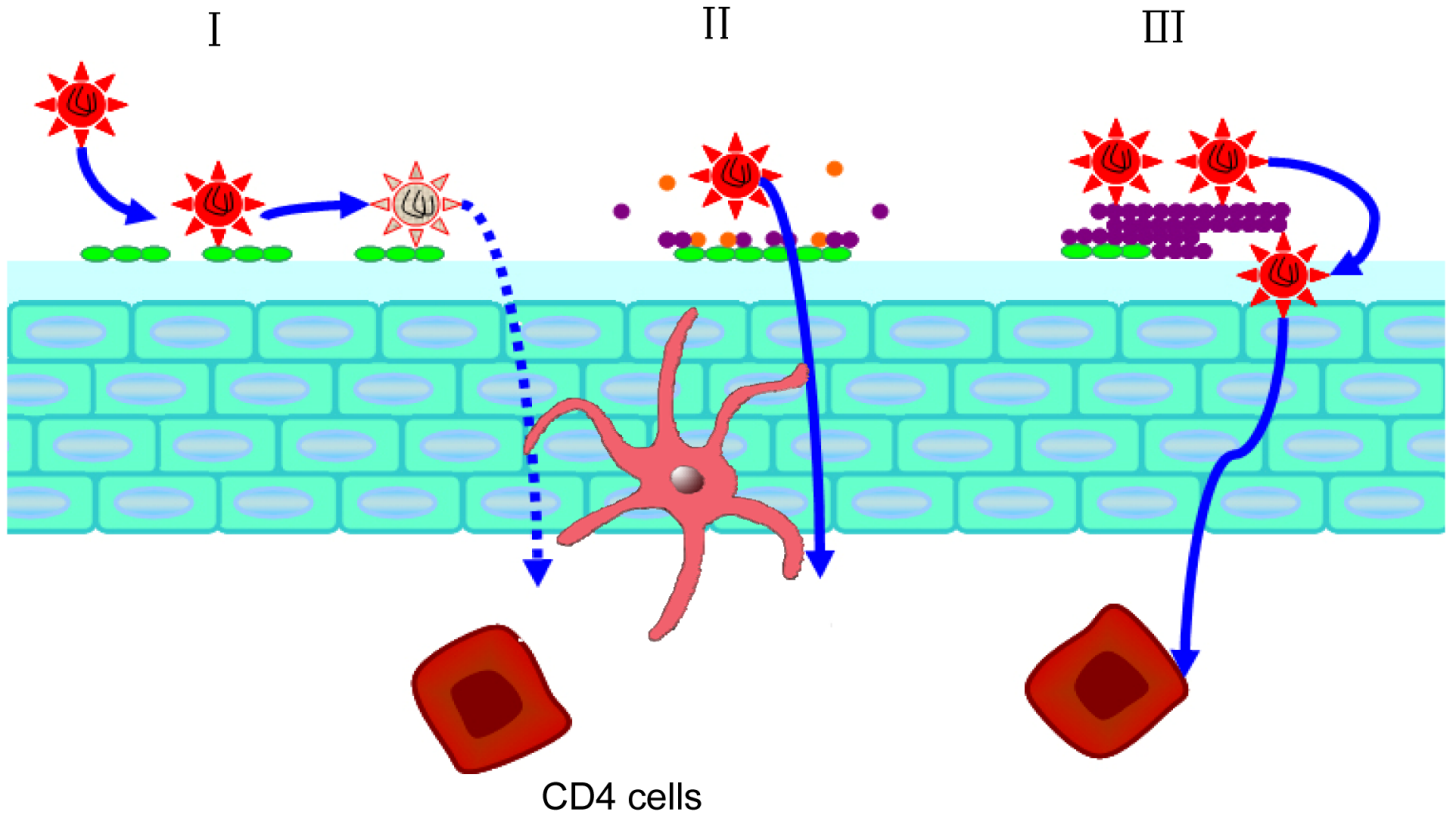
Schematic presentation of drug-protein interaction that result in the poor efficacy of polyanionic candidate microbicides in clinical trials. Prior to the sexual intercourse, candidate microbicide was intravaginally applied and these anionic macromolecules remained on the vaginal surface rather than distributed in the inner tissue. When the viruses arrived, polyanions neutralized the viruses and stopped HIV at the gate (I). However, during sexual intercourse, viruses were delivered in semen, in which cationic peptides/proteins competitively interacted with polyanions so that there might not be sufficient polyanions to interfere with HIV-1 infection (II). Even worse, the interaction between polyanions and PAP derived fragments led to a facilitated process of amyloid fibril formation. The accelerated amyloid fibril formation increased the risk of HIV sexual transmission (III). Green particles, polyanionic molecules, purple particles, PAP derived fragments, orange particles, non-PAP derived cationic peptides/proteins.

As expected, our result is consistent with the clinical outcomes. None of the Phase III clinical trials of polyanions has shown protection against HIV-1 transmission. In some cases, viral infections have even been enhanced. Such loss of antiviral activity might result from the neutralization effect of the cationic seminal proteins on the negative charges of the polyanions. However, the promotion of SE-F-derived amyloid fibril formation with polyanions might be the reason for the increased risk of viral acquisition (Fig. 10). Although not every clinical trial of polyanion showed enhancement of viral transmission, sexual intercourse is a complicated event influenced by an array of factors, such as the frequency, intensity or duration of the sexual intercourse, as well as ejaculate volume or composition. Moreover, the vaginal tract appears a relatively large surface area of about 100 cm^2^ [51]. Prior to the sexual intercourse, vaginal gel was spread on the surface of vaginal tract. The homogeneity of the applied gel might also determine the interaction between drugs and seminal proteins. Since SEVI enhance HIV-1 infection by promoting the process of viral binding to the cells, sufficient polyanions are required to exert the anti-HIV effect during sexual intercourse. Therefore, all polyanions that entered the Phase III efficacy trials showed general poor efficacy against HIV-1 sexual transmission. Nevertheless, the failure of the clinical trials of polyanion-based microbicides may be caused by multiple factors, such as the polyanion-induced inflammatory reactions in the mucosa, the ineffectiveness of some polyanions against HIV-1 R5 strains, and low adherence in using microbicides before or during the sexual acts, etc [14]–[17]. Therefore, polyanion-facilitated formation of SEVI amyloid fibrils may be considered as one of the causes for the failure of the polyanion-based microbicide clinical trial.

In conclusion, our results demonstrated that polyanion-based microbicides are able to accelerate the formation of SE-F-derived amyloid fibrils by electrostatic interaction, resulting in aggravated enhancement of HIV-1 infection, which may be one of the causes of their failure in clinical trials. As such, drug and semen interaction should be one of the key factors in evaluating the efficacy of newly proposed candidate microbicides, and such candidates should proceed to human clinical trials only after having been fully defined with respect to their pharmacokinetics and pharmacodynamics profiles in semen, especially those macromolecules that could not be absorbed into the inner vaginal tissues. Since other report showed that polyanions might attenuate the enhancement effect of SEVI on HIV infection due to their negatively charged properties, polyanions might be developed as part as a combination microbicide strategy rather than a monotherapy.

## References

[1] Belec L, Jenabian MA, Charpentier C, Saidi H (2011) Combinatorial prevention of HIV transmission in women: the case for a vaginal microbicide. Future Microbiol 6: 731–737. 21797688 10.2217/fmb.11.64

[2] Wood H, Colver H, Stewart E, Palfreeman A (2011) Increasing uptake of HIV tests in men who have sex with men. Practitioner 255: 25–8, 3. 21789986

[3] McGowan I (2008) Rectal microbicides: a new focus for HIV prevention. Sex Transm Infect 84: 413–417. 19028937 10.1136/sti.2008.031328

[4] McGowan I, Anton P (2008) Rectal microbicides. Curr Opin HIV AIDS 3: 593–598. 19373027 10.1097/COH.0b013e32830891cf

[5] Abdool KQ, Abdool Karim SS, Frohlich JA, Grobler AC, Baxter C, et al (2010) Effectiveness and safety of tenofovir gel, an antiretroviral microbicide, for the prevention of HIV infection in women. Science 329: 1168–1174. 20643915 10.1126/science.1193748 PMC3001187

[6] Moulard M, Lortat-Jacob H, Mondor I, Roca G, Wyatt R, et al (2000) Selective interactions of polyanions with basic surfaces on human immunodeficiency virus type 1 gp120. J Virol 74: 1948–1960. 10644368 10.1128/jvi.74.4.1948-1960.2000 PMC111673

[7] Christensen ND, Reed CA, Culp TD, Hermonat PL, Howett MK, et al (2001) Papillomavirus microbicidal activities of high-molecular-weight cellulose sulfate, dextran sulfate, and polystyrene sulfonate. Antimicrob Agents Chemother 45: 3427–3432. 11709319 10.1128/AAC.45.12.3427-3432.2001 PMC90848

[8] Simoes JA, Citron DM, Aroutcheva A, Anderson RA Jr, Chany CJ, et al (2002) Two novel vaginal microbicides (polystyrene sulfonate and cellulose sulfate) inhibit Gardnerella vaginalis and anaerobes commonly associated with bacterial vaginosis. Antimicrob Agents Chemother 46: 2692–2695. 12121959 10.1128/AAC.46.8.2692-2695.2002 PMC127353

[9] Van DL, Govinden R, Mirembe FM, Guedou F, Solomon S, et al (2008) Lack of effectiveness of cellulose sulfate gel for the prevention of vaginal HIV transmission. N Engl J Med 359: 463–472. 18669425 10.1056/NEJMoa0707957

[10] Halpern V, Ogunsola F, Obunge O, Wang CH, Onyejepu N, et al (2008) Effectiveness of cellulose sulfate vaginal gel for the prevention of HIV infection: results of a Phase III trial in Nigeria. PLoS ONE 3: e3784. 19023429 10.1371/journal.pone.0003784 PMC2582655

[11] Skoler-Karpoff S, Ramjee G, Ahmed K, Altini L, Plagianos MG, et al (2008) Efficacy of Carraguard for prevention of HIV infection in women in South Africa: a randomised, double-blind, placebo-controlled trial. Lancet 372: 1977–1987. 19059048 10.1016/S0140-6736(08)61842-5

[12] Abdool Karim SS (2010) Results of effectiveness trials of PRO 2000 gel: lessons for future microbicide trials. Future Microbiol 5: 527–529. 20353292 10.2217/fmb.10.29

[13] McCormack S, Ramjee G, Kamali A, Rees H, Crook AM, et al (2010) PRO2000 vaginal gel for prevention of HIV-1 infection (Microbicides Development Programme 301): a phase 3, randomised, double-blind, parallel-group trial. Lancet 376: 1329–1337. 20851460 10.1016/S0140-6736(10)61086-0 PMC2956883

[14] Cutler B, Justman J (2008) Vaginal microbicides and the prevention of HIV transmission. Lancet Infect Dis 8: 685–697. 18992405 10.1016/S1473-3099(08)70254-8 PMC2627483

[15] Mesquita PM, Cheshenko N, Wilson SS, Mhatre M, Guzman E, et al (2009) Disruption of tight junctions by cellulose sulfate facilitates HIV infection: model of microbicide safety. J Infect Dis 200: 599–608. 19586414 10.1086/600867 PMC2877491

[16] Morris GC, Lacey CJ (2010) Microbicides and HIV prevention: lessons from the past, looking to the future. Curr Opin Infect Dis 23: 57–63. 19918175 10.1097/QCO.0b013e328334de6d

[17] Tao W, Richards C, Hamer D (2008) Enhancement of HIV infection by cellulose sulfate. AIDS Res Hum Retroviruses 24: 925–929. 18627218 10.1089/aid.2008.0043 PMC2927036

[18] Pirrone V, Wigdahl B, Krebs FC (2011) The rise and fall of polyanionic inhibitors of the human immunodeficiency virus type 1. Antiviral Res 90: 168–182. 21439325 10.1016/j.antiviral.2011.03.176

[19] Bourne N, Bernstein DI, Ireland J, Sonderfan AJ, Profy AT, et al (1999) The topical microbicide PRO 2000 protects against genital herpes infection in a mouse model. J Infect Dis 180: 203–205. 10353881 10.1086/314853

[20] Patel S, Hazrati E, Cheshenko N, Galen B, Yang H, et al (2007) Seminal plasma reduces the effectiveness of topical polyanionic microbicides. J Infect Dis 196: 1394–1402. 17922405 10.1086/522606

[21] Neurath AR, Strick N, Li YY (2006) Role of seminal plasma in the anti-HIV-1 activity of candidate microbicides. BMC Infect Dis 6: 150. 17042959 10.1186/1471-2334-6-150 PMC1618840

[22] Munch J, Rucker E, Standker L, Adermann K, Goffinet C, et al (2007) Semen-derived amyloid fibrils drastically enhance HIV infection. Cell 131: 1059–1071. 18083097 10.1016/j.cell.2007.10.014

[23] Roan NR, Munch J, Arhel N, Mothes W, Neidleman J, et al (2009) The cationic properties of SEVI underlie its ability to enhance human immunodeficiency virus infection. J Virol 83: 73–80. 18945786 10.1128/JVI.01366-08 PMC2612336

[24] Monsellier E, Ramazzotti M, Taddei N, Chiti F (2010) A computational approach for identifying the chemical factors involved in the glycosaminoglycans-mediated acceleration of amyloid fibril formation. PLoS ONE 5: e11363. 20613870 10.1371/journal.pone.0011363 PMC2894048

[25] Cohlberg JA, Li J, Uversky VN, Fink AL (2002) Heparin and other glycosaminoglycans stimulate the formation of amyloid fibrils from alpha-synuclein in vitro. Biochemistry 41: 1502–1511. 11814343 10.1021/bi011711s

[26] McLaurin J, Franklin T, Zhang X, Deng J, Fraser PE (1999) Interactions of Alzheimer amyloid-beta peptides with glycosaminoglycans effects on fibril nucleation and growth. Eur J Biochem 266: 1101–1110. 10583407 10.1046/j.1432-1327.1999.00957.x

[27] Castillo GM, Ngo C, Cummings J, Wight TN, Snow AD (1997) Perlecan binds to the beta-amyloid proteins (A beta) of Alzheimer's disease, accelerates A beta fibril formation, and maintains A beta fibril stability. J Neurochem 69: 2452–2465. 9375678 10.1046/j.1471-4159.1997.69062452.x

[28] Ren R, Hong Z, Gong H, Laporte K, Skinner M, et al (2010) Role of glycosaminoglycan sulfation in the formation of immunoglobulin light chain amyloid oligomers and fibrils. J Biol Chem 285: 37672–37682. 20870723 10.1074/jbc.M110.149575 PMC2988372

[29] Suk JY, Zhang F, Balch WE, Linhardt RJ, Kelly JW (2006) Heparin accelerates gelsolin amyloidogenesis. Biochemistry 45: 2234–2242. 16475811 10.1021/bi0519295 PMC2657342

[30] Bourgault S, Solomon JP, Reixach N, Kelly JW (2011) Sulfated glycosaminoglycans accelerate transthyretin amyloidogenesis by quaternary structural conversion. Biochemistry 50: 1001–1015. 21194234 10.1021/bi101822y PMC3035766

[31] Calamai M, Kumita JR, Mifsud J, Parrini C, Ramazzotti M, et al (2006) Nature and significance of the interactions between amyloid fibrils and biological polyelectrolytes. Biochemistry 45: 12806–12815. 17042499 10.1021/bi0610653

[32] Sackett K, Wexler-Cohen Y, Shai Y (2006) Characterization of the HIV N-terminal fusion peptide-containing region in context of key gp41 fusion conformations. J Biol Chem 281: 21755–21762. 16751188 10.1074/jbc.M603135200

[33] Jiang S, Lu H, Liu S, Zhao Q, He Y, Debnath AK (2004) N-substituted pyrrole derivatives as novel human immunodeficiency virus type 1 entry inhibitors that interfere with the gp41 six-helix bundle formation and block virus fusion. Antimicrob Agents Chemother 48: 4349–4359. 15504864 10.1128/AAC.48.11.4349-4359.2004 PMC525433

[34] Lu H, Zhao Q, Wallace G, Liu S, He Y, et al (2006) Cellulose acetate 1,2-benzenedicarboxylate inhibits infection by cell-free and cell-associated primary HIV-1 isolates. AIDS Res Hum Retroviruses 22: 411–418. 16706617 10.1089/aid.2006.22.411 PMC2788998

[35] Li L, Qiao P, Yang J, Lu L, Tan S, et al (2010) Maleic anhydride-modified chicken ovalbumin as an effective and inexpensive anti-HIV microbicide candidate for prevention of HIV sexual transmission. Retrovirology 7: 37. 20420669 10.1186/1742-4690-7-37 PMC2888735

[36] Lacey CJ, Woodhall S, Qi Z, Sawant S, Cowen M, et al (2010) Unacceptable side-effects associated with a hyperosmolar vaginal microbicide in a phase 1 trial. Int J STD AIDS 21: 714–717. 21139151 10.1258/ijsa.2010.010215

[37] Bourne N, Zaneveld LJ, Ward JA, Ireland JP, Stanberry LR (2003) Poly(sodium 4-styrene sulfonate): evaluation of a topical microbicide gel against herpes simplex virus type 2 and Chlamydia trachomatis infections in mice. Clin Microbiol Infect 9: 816–822. 14616702 10.1046/j.1469-0691.2003.00659.x

[38] Herold BC, Bourne N, Marcellino D, Kirkpatrick R, Strauss DM, et al (2000) Poly(sodium 4-styrene sulfonate): an effective candidate topical antimicrobial for the prevention of sexually transmitted diseases. J Infect Dis 181: 770–773. 10669374 10.1086/315228

[39] Kim KA, Yolamanova M, Zirafi O, Roan NR, Staendker L, et al (2010) Semen-mediated enhancement of HIV infection is donor-dependent and correlates with the levels of SEVI. Retrovirology 7: 55. 20573198 10.1186/1742-4690-7-55 PMC2914040

[40] Garg S, Goldman D, Krumme M, Rohan LC, Smoot S, et al (2010) Advances in development, scale-up and manufacturing of microbicide gels, films, and tablets. Antiviral Res 88 Suppl 1: S19–S29. 21109064 10.1016/j.antiviral.2010.09.010

[41] Belec L, Jenabian MA, Charpentier C, Saidi H (2011) Combinatorial prevention of HIV transmission in women: the case for a vaginal microbicide. Future Microbiol 6: 731–737. 21797688 10.2217/fmb.11.64

[42] Nilsson MR (2004) Techniques to study amyloid fibril formation in vitro. Methods 34: 151–160. 15283924 10.1016/j.ymeth.2004.03.012

[43] Epstein EA, Chapman MR (2008) Polymerizing the fibre between bacteria and host cells: the biogenesis of functional amyloid fibres. Cell Microbiol 10: 1413–1420. 18373633 10.1111/j.1462-5822.2008.01148.x PMC2674401

[44] Motamedi-Shad N, Monsellier E, Chiti F (2009) Amyloid formation by the model protein muscle acylphosphatase is accelerated by heparin and heparan sulphate through a scaffolding-based mechanism. J Biochem 146: 805–814. 19675100 10.1093/jb/mvp128

[45] Solomon JP, Bourgault S, Powers ET, Kelly JW (2011) Heparin binds 8 kDa gelsolin cross-beta-sheet oligomers and accelerates amyloidogenesis by hastening fibril extension. Biochemistry 50: 2486–2498. 21348501 10.1021/bi101905n PMC3068913

[46] Martellini JA, Cole AL, Svoboda P, Stuchlik O, Chen LM, et al (2011) HIV-1 enhancing effect of prostatic acid phosphatase peptides is reduced in human seminal plasma. PLoS ONE 6: e16285. 21283773 10.1371/journal.pone.0016285 PMC3024420

[47] Roan NR, Muller JA, Liu H, Chu S, Arnold F, et al (2011) Peptides Released by Physiological Cleavage of Semen Coagulum Proteins Form Amyloids that Enhance HIV Infection. Cell Host Microbe 10: 541–550. 22177559 10.1016/j.chom.2011.10.010 PMC3257029

[48] Scordi-Bello IA, Mosoian A, He C, Chen Y, Cheng Y, et al (2005) Candidate sulfonated and sulfated topical microbicides: comparison of anti-human immunodeficiency virus activities and mechanisms of action. Antimicrob Agents Chemother 49: 3607–3615. 16127029 10.1128/AAC.49.9.3607-3615.2005 PMC1195443

[49] Venkataraman N, Cole AL, Svoboda P, Pohl J, Cole AM (2005) Cationic polypeptides are required for anti-HIV-1 activity of human vaginal fluid. J Immunol 175: 7560–7567. 16301665 10.4049/jimmunol.175.11.7560

[50] Martellini JA, Cole AL, Venkataraman N, Quinn GA, Svoboda P, et al (2009) Cationic polypeptides contribute to the anti-HIV-1 activity of human seminal plasma. FASEB J 23: 3609–3618. 19487309 10.1096/fj.09-131961 PMC3236594

[51] Pendergrass PB, Belovicz MW, Reeves CA (2003) Surface area of the human vagina as measured from vinyl polysiloxane casts. Gynecol Obstet Invest 55: 110–113. 12771458 10.1159/000070184

